# Influences of Biodynamic and Conventional Farming Systems on Quality of Potato (*Solanum Tuberosum* L.) Crops: Results from Multivariate Analyses of Two Long-Term Field Trials in Sweden

**DOI:** 10.3390/foods4030440

**Published:** 2015-09-15

**Authors:** Lars Kjellenberg, Artur Granstedt

**Affiliations:** 1 Department of Plant Breeding, Swedish University of Agriculture, P.O. Box 101, SE 230 53 Alnarp, Sweden; 2 Biodynamic Research Institute, Skilleby, SE 153 91 Järna, Sweden; E-Mail: arturgranstedt@jdb.se

**Keywords:** biodynamic agriculture, conventional agriculture, product quality, principal component analysis, potato

## Abstract

The aim of this paper was to present results from two long term field experiments comparing potato samples from conventional farming systems with samples from biodynamic farming systems. The principal component analyses (PCA), consistently exhibited differences between potato samples from the two farming systems. According to the PCA, potato samples treated with inorganic fertilizers exhibited a variation positively related to amounts of crude protein, yield, cooking or tissue discoloration and extract decomposition. Potato samples treated according to biodynamic principles, with composted cow manure, were more positively related to traits such as Quality- and EAA-indices, dry matter content, taste quality, relative proportion of pure protein and biocrystallization value. Distinctions between years, crop rotation and cultivars used were sometimes more significant than differences between manuring systems. Grown after barley the potato crop exhibited better quality traits compared to when grown after ley in both the conventional and the biodynamic farming system.

## 1. Introduction

Are there differences between produce from Organic Agriculture (OA) in comparison to the corresponding produce originating from Conventional Agriculture (CA)? Ever since the dawn of OA almost one hundred years ago, this question has attained a great deal of attention. Several reviews have been published on the topic [1,2,3,4,5,6,7,8,9,10,11]. Despite these efforts, the question of the quality of food originating from OA still remains open. One reason for this is perhaps that comparing farming systems is a complex task, and often demands several years until significant differences appear.

Biodynamic Agriculture (BA) is one of the oldest OA farming systems. Its roots go back to the middle of the nineteen twenties. More details on BA can be found elsewhere [12,13,14,15,16]. Like other OA systems, BA relies on nitrogen fixation by leguminoses, a crop rotation comprising more than four seasons and the use of organic, mostly composted, manure. What makes BA different to most other OA is its spiritual approach to agriculture [17] and special biodynamic preparations used both in the manure and on the field [18,19]. Being one of the oldest, and at the same time a somewhat controversial OA system, has caused BA to be included in several studies and field trials [1,20,21,22,23,24,25,26,27,28,29,30,31,32,33,34].

In 1958, the Scandinavian Research Circle on Biodynamic Agriculture (SRC) in Järna, Sweden, began an agricultural field experiment that lasted until 1990, *i.e.*, 33 years. The field experiment, called the K-trial, included eight different fertilizer treatments. Various quality parameters and quality-assessment methods were developed and tested during the experimental period [35,36,37].

From the K-trial, another long term field experiment emerged. This so called UJ-trial was a cooperation between the SRC and the Swedish University of Agriculture. It ran during 1971–1976 in Uppsala, and 1971–1979 in Järna, at a location very close to the K-trial. In the UJ- trial comparisons were made between two farming systems; biodynamic farming and conventional farming, in which both fertilizer regimes and crop rotations were studied. So far, mainly reports in Swedish have been published from the UJ-trial [38,39].

During the whole period, from 1958 to 1990, Agr.lic. Bo D. Pettersson was responsible for the practical and analytical work connected to the two field trials. The fact that the same person is performing most of the work on the same field trials for 33 years is probably quite unique. It also diminishes the influences from the human factor, that otherwise probably contributes to the variations within the results from long term field experiments.

Since the termination of the K- and UJ-trial, the results from similar long-term field trial have been published [1,20,40,41]. Results in English from the two field trials at the SRC have not yet been covered in scientific papers. References to the primary data included in the principal component analyses (PCA) of this paper are therefore mainly published in Swedish [38,42].

The aim of this paper is to present and discuss results, concerning potato samples, from these two long term field experiments comparing CA and BA. This paper is also a tribute to Bo D. Pettersson, one of the pioneers in Scandinavia concerning research on BA.

## 2. Material and Methods

Both the K-trial and the UJ field experiments were located approximately 700 m apart at 59° N, 17° E and at an elevation of 10 m above sea level. The mean yearly precipitation was 550 mm, and the mean annual temperature was 6 °C, with 6–8 snow-free months per year.

### 2.1. Design of the K-Trial

The design of the K-trial included eight different fertilizer treatments without repetitions. The size of each subplot was 36 m². The following crops were rotated without interruption; summer wheat (under sown with clover/grass), clover/grass ley, potatoes and beets. The soil was a silty loam with intermediate humus content. From the K-trial, this paper includes a comparison between biodynamic manuring (K1) and conventional inorganic fertilizers (K8). Applied amounts of nutrients to different crops in the rotation are displayed in Table 1. Neither herbicides nor pesticides were used in the K-trial. Cultivars used were: 1970–1973 King Edward, 1974–1981 Grata, 1982–1985 Bellona, 1986–1989 Provita.

**Table 1:** Average supply of N, P and K in kg/ha and year to different crops in the different treatments of the K-trial 1970–1989.

	Nutrient, kg/ha					
	N		P		K	
Crop	K1	K8	K1	K8	K1	K8
Ley	0	0	0	0	0	0
Potatoes	128 ^1^	187 ^2^	61 ^1^	86 ^2^	122 ^1^	129 ^2^
Beets	192 ^1^	187 ^3^	91 ^1^	58 ^3^	182 ^1^	195 ^3^
Wheat	0	94 ^4^	0	0	0	0

0 = not manured; ^1^ Composted cow manure supplemented with bone meal. Biodynamic preparations; ^2^ NPK inorganic fertilizer with trace elements; ^3^ NPK inorganic fertilizer with trace elements + N inorganic fertilizer; ^4^ N inorganic fertilizer.

### 2.2. Design of the UJ-Trial

In the UJ experiment, conventional (A) and biodynamic (B) farming systems were compared with each other, in two crop rotations: A1 and B1 represented a three-year rotation without ley (potato-wheat-barley), whereas A2 and B2 represented a three-year rotation with ley (ley-potatoes-wheat). All crops were grown each year. The soil was a silty loam, well supplied with plant nutrients, but with low humus content. The UJ-trial was divided into two parts, the main UJ-trial and the supplementary UJ-trial.

#### 2.2.1. The Main UJ-Trial

In the main UJ-trial a split-split-plot design was used with three replications. The split factor was crop rotation. Applied amounts of nutrients are displayed in Table 2.

**Table 2:** Average supply of N, P and K in kg/ha and year, to different crops in the UJ-trial.

Nutrient, kg/ha	UJ-Trial (Two 3 Year Crop Rotations)				
	Crop	A1 ^1^	A2 ^1^	B1 ^2^	B2 ^2^
N	Ley	-	0	-	0
	Potatoes	120	100	120	100
	Wheat	80	40	70	50
	Barley	80	-	60	-
P	Ley	-	0	-	0
	Potatoes	57	80	85	70
	Wheat	38	32	49	35
	Barley	38	-	42	-
K	Ley	-	0	-	0
	Potatoes	139	190	106	90
	Wheat	93	76	62	45
	Barley	93	-	53	-

0 = not manured; - = not part of the crop rotation; ^1^ NPK inorganic fertilizer with trace elements; ^2^ Composted cow manure supplemented with bone meal and biodynamic compost preparations.

The main UJ-trial consisted of 36 plots; the total size of each plot having a size of 12 × 12 m.

#### 2.2.2. The Additional UJ-Trial

Within the frames of the UJ-trial, a supplementary study was performed along one of the repetitions of the A2 and B2 plots in the main trial. This additional UJ-trial was designed for comparing the effect of increasing amounts of fertilizers. Applied amounts of nutrients, to this additional study are shown in Table 3. The proportions between N, P and K, in the two types of fertilizers, were the same as in the main trial. Herbicides and pesticides were used only in A2. Cultivar used was Bintje. The additional UJ-trial was performed 1971 to 1974.

**Table 3:** Type and amounts of nitrogen, kg N/ha, given to potato, in the additional UJ-trial, 1971–1974.

Type	Amounts of Nitrogen, kg N/ha					
Inorganic NPK-fertilizer	0	40	80	120	160	200
Biodynamic compost	0	63	126	189	252	315

The additional UJ-trial consisted of 36 plots without repetitions; the total size of each plot was 5 × 4 m.

### 2.3. Analyses

The analyses used in connection with the K-trial and both the main and the additional UJ-trial are described more in detail elsewhere [35,38,43]. In short, the following analyses were used in the principal component analyses (the short designation of each analysis used in the figures are written in italics):

*Amino acids:* The total amounts of amino acids, expressed as % of crude protein, were determined by chromatography according to Moore and Stein (1963) [44].

*Ascorbic acid*: The amounts of ascorbic acid were determined by titration with 2,6-dichlorphenolindophenol as indicator.

*Big*: Relative proportion of potato tubers bigger than 50 mm.

*Branching*: The number of main stems and lateral stems over 10 cm long were recorded on 20 plants/plot, shortly after blossoming. With the aid of regression analyses, the number of lateral stems was adjusted to an equal number of main stems, in order to make the results comparable.

*Cook Apr and Cook Dec*: The discoloration occurring in cooked potatoes was measured on 100 tubers. 50 tubers were cooked with skins and the other half without, prior to the darkening examination. The results were given as the average discoloration from the two groups. This analysis was performed twice a year, once on newly harvested samples (Cook Dec) and secondly after 6 months of storage (Cook Apr).

*Crude*: Crude protein: The total N-content was determined by the Kjeldahl-method. The crude protein content was calculated according to standard values.

*Crystal*: Biocrystallization analysis: The method was performed by letting an organic extract influence the crystallization of copper chloride on a glass plate [45,46]. The evaluation of the various sections of the plates was performed on the basis of a pre-established scale according to Pettersson (1970) [43]. High values were supposed to indicate a higher structural quality.

*Decompos*: Extract decomposition: A tissue extract of the potato was allowed to stand at 20 °C. The decomposition was recorded by daily measurements of the electrical resistance (alternatively conductance) in the extract. The maximal change in resistance, Rd, was compared to the initial value, R0, and the quotient expressed as a percent value (100% Rd/R0).

*Dry matter*: Determination of the amount of dry matter in relation to total fresh tuber weight was performed according to standard.

*EAA-index*: Determination of the abundance of different amino acids was made according to Oser (1951) [47]. The following eight amino acids were used in the calculations, lysine, methionine, leucine, threonine, isoleucine, tryptophan, valine and phenylalanine. The relative composition of the same amino acids in egg was used as a reference.

*Extract*: The darkening of potato extract was measured daily for four days. The measurements were performed with a spectrophotometer at 530 nm. The angle coefficient, Eb, or slope of the absorbance curve was applied as criterion for the speed of darkening. The higher the Eb-value, the faster was the darkening in the potato extracts.

*Free amino ac*: The Sorensen formal-method with titration to pH 8.5 was used to determine the amounts of free amino acids. The amount of free amino acids was expressed as mg N/100 g dry matter.

*K-index*: An integrated value of the results from three separate analyses; with the ratio value in parenthesis: Free amino (275), Extract (500), Decomposition (25). The obtained value from a separate method was expressed in % of the equivalent ratio value and then inverted arithmetically around the value 100. The three values are added and the sum is divided by three. The obtained value was described as the Quality-indices, according to Pettersson [43].

*Losses*: Amount of potato tubers lost during storage in percent of amount tubers put into storage.

*Phyt*: The strength of the Late blight (*Phytophtora infestans*) attack was graded, several times per season, on each plant, on the entire row, and finally on the entire plot. In the PCA, the annual mean value of the attack was used.

*Pure*: The level of crude *versus* true protein was first determined on the basis of Kjeldahl-N, and then the true protein was precipitated with Cu2S04 and NaOH and expressed as percent of dry matter.

*Rel. pure*: The relative amount of pure protein was expressed as per cent of crude protein.

*Remains*: The remaining total tuber yield in April, after storage.

*Sorted*: The field yield corrected after first sorting and adjusted to 20% dry matter content.

*Small*: Relative proportion of potato tubers smaller than 50 mm.

*Taste Apr and Taste Dec*: The flavor of the potatoes was examined shortly after harvest (December) and at the end (April) of the storage period. For each test, 50 medium-sized tubers were used to arrive at the flavor value or taste-point, expressed in a scale of 1–4 where 1 = poorest and 4 = best flavor.

*Tissue*: The enzymatic darkening of raw exposed potato tissue was measured with a reflectance attachment on a photo spectrometer. Sliced tubers, allowed to darken naturally for two days under controlled high-humidity conditions, were compared in their reflectance with freshly-sliced, undarkened tubers. The darkening was expressed as per cent change over the initial value.

*Yield*: Yield, on field at harvest occasion, unsorted tuber weight.

### 2.4. Treatment of Data

The computer programs; Excel 2010 (Microsoft Corp., Redmond, WA, USA), Minitab 16.0 (Minitab Inc., State College, PA, USA) and SPSS 16.0 (IBM Inc., Armonk, NY, USA) were used for calculations and statistical evaluations.

### 2.5. Principle Component Analyses

**Figure 1:**
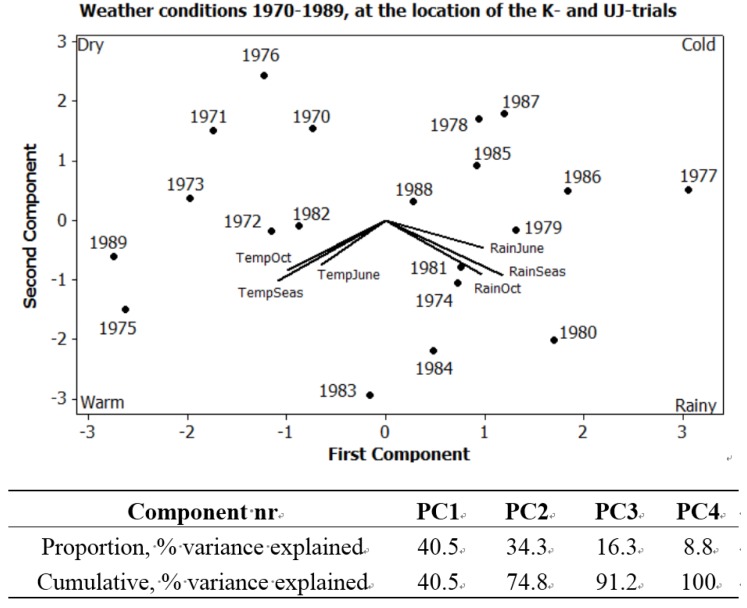
PCA plot on weather conditions at location of field trials 1970–1989, TempJune = Daily temperature sum from 1 April to 30 June, TempOct = Daily temperature sum from 1 July to 31 October, TempSeas = Daily temperature sum from 1 April to 31 October, RainJune = Sum of precipitation from 1 April to 30 June, RainOct = Sum of precipitation from 1 July to 31 October, RainSeas = Sum of precipitation from 1 April to 31 October.

**Figure 2:**
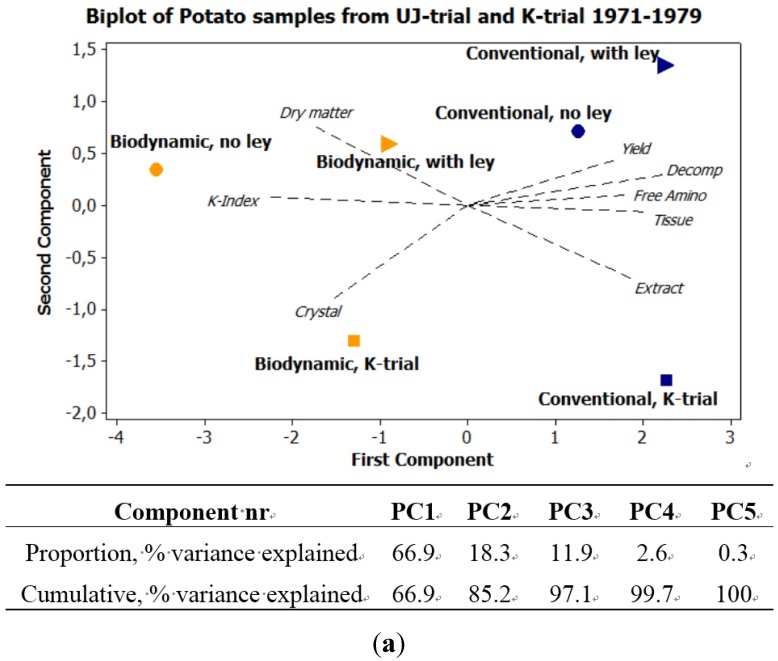

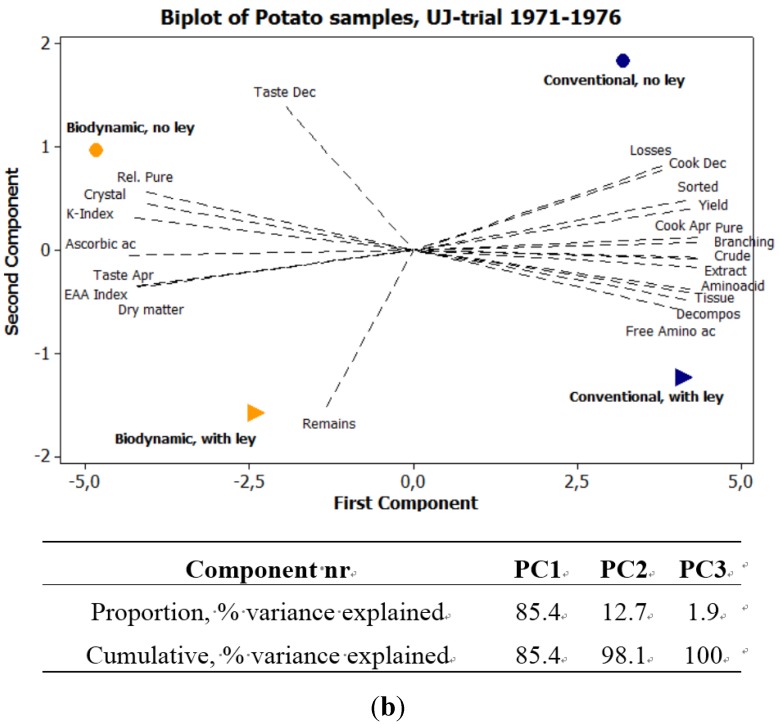
PCA plots on potato samples from the K- and UJ-trial. (**a**) Mean values of the 8 core variables used both in the UJ-trial and in the K-trial 1971–1979; (**b**) Mean values of the 22 variables used in the UJ-trial 1971 to 1976.

**Figure 3:**
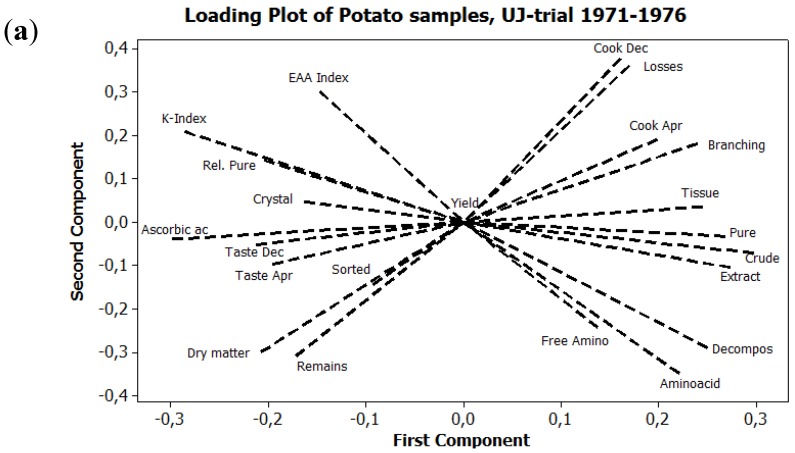

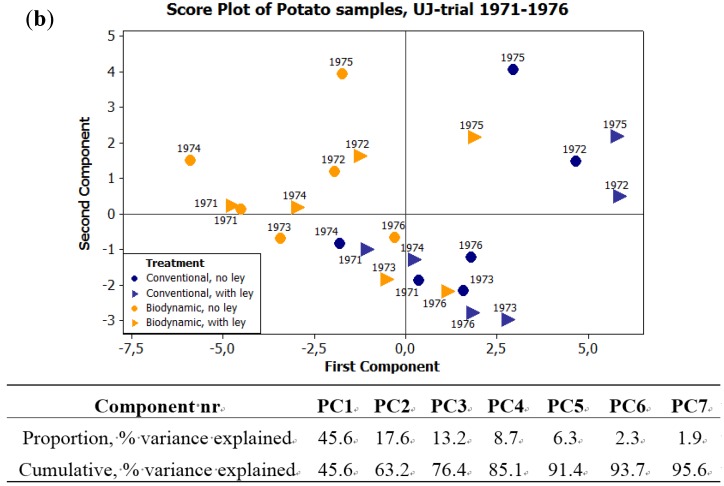
PCA plots on annual, mean values of potato samples in the UJ-trial 1971–1976. (**a**) Loading plot on 22 different variables; (**b**) Score plot expressing the variation among different treatments and seasons. Cultivar Bintje.

**Figure 4:**
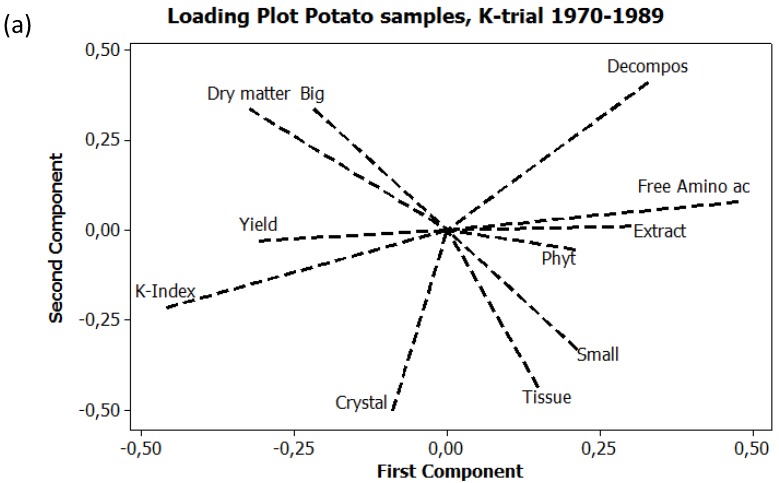

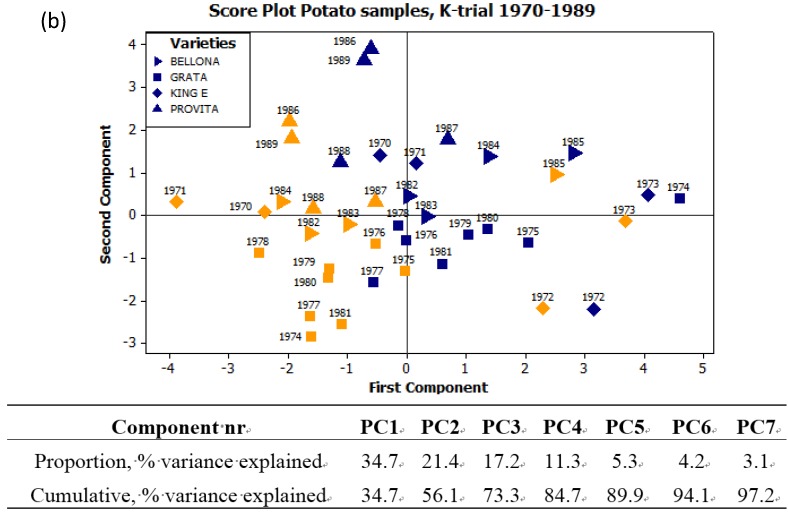
PCA plots on potato samples from the K-trial 1970–1989. (**a**) Loading plot of all common 11 analyses; (**b**) Score plot expressing manuring systems, cultivars and seasons. Cultivars: 1970–1973 King Edward, 1974–1981 Grata, 1982–1985 Bellona, 1986–1989 Provita. Orange symbols: biodynamic compost, blue symbols; conventional inorganic fertilizer.

**Figure 5:**
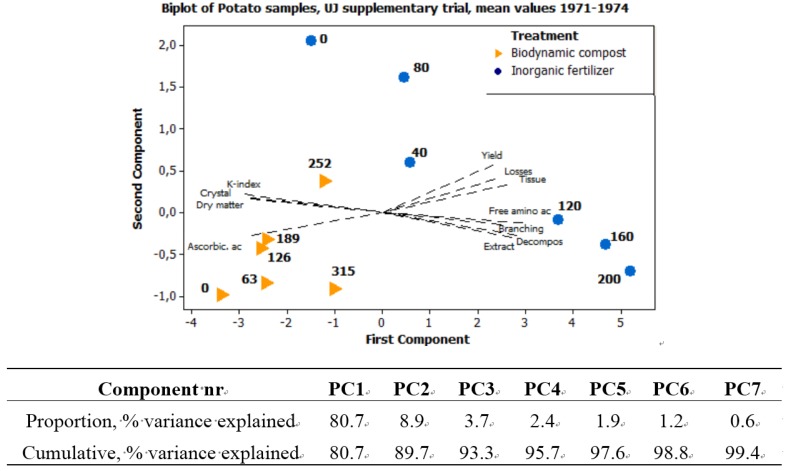
PCA plot of mean values from potato samples are made into manure with different amounts of fertilizers, in the additional UJ trial 1971–1974. Bolded numbers indicate amounts of nitrogen applied, kg N/ha.

**Figure 6:**
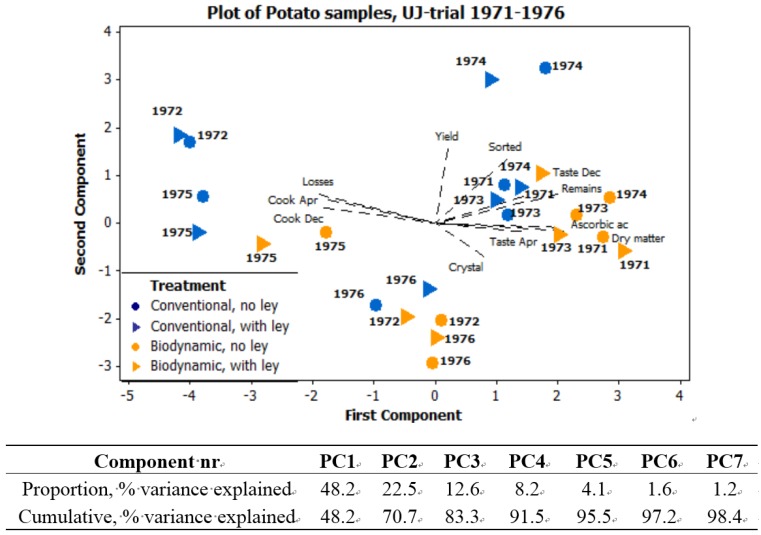
PCA plot of mean values, without the core variables and other variables closely correlated to the core variables, from potato samples. UJ-trial 1971–1976. Bolded numbers indicate harvest year.

Principal component analysis (PCA) was used, with a correlation matrix, to describe interactions between subjects. Six different PCA are presented here. Figure 1 exhibits the seasonal variations in precipitation and temperature, 1970–1989, using six weather variables. Figure 2a expresses the mean values of potato samples 1971–1979 from the eight core parameters used both in the main UJ- and in the K-trial. Figure 2b displays the mean values and Figure 3, the annual values, from the main UJ-trial 1971–1976 using 22 different variables. Figure 4 shows the annual results from 11 parameters examined each year 1970–1989 in the K-trial. Figure 5 is the only PCA using samples from the supplementary UJ-trial; including the mean values of 11 different variables with potato samples from 1971–1974. Figure 6 displays a PCA with mean annual values from 11 parameters in the main UJ-trial but without the eight core variables used in all the other PCA.

### 2.6. Weather Conditions

Data on temperature and precipitation 1970–1989 were obtained from a weather station at the field trial and are expressed in Figure 1. The seasons of 1980, 1983 and 1984 were warm and rainy, the season of 1975 was warm and dry, the seasons of 1977 and 1987 were cold and rainy, whereas the season of 1976 was cold and dry (Figure 1).

## 3. Results

The PCA of the potato samples exhibited correlations between variables and differences between treatments, seasons, crop rotation and cultivars.

The PCA plots allowed mainly the following comparisons between subjects:

- **Figure 2a**, differences between field trials;
- **Figure 2a,b and Figure 3**, differences between farming systems;
- **Figure 4 and Figure 5**, differences between type and amount of manure;
- **Figure 3 and Figure 4**, differences between seasons or cultivars;
- **Figure 6**, differences in the PCA plot depending on the selection of variables.

### 3.1. Relations between Variables

A total of 23 different variables were used in the PCA presented here. A core of eight variables was used in all PCA, except in Figure 6. These eight were; biocrystallization (Crystal), dry matter content (Dry matter), Quality Indices (K-index), yield (Yield) extract decomposition (Decompos), free amino acids (Free amino ac), extract discoloration (Extract) and tissue darkening (Tissue).

The variances between the variables exhibited a similar pattern in all PCA. Two clusters of variables appeared along the first principal component.

The first cluster was formed by the four core variables; free amino acids, extract decomposition, extract discoloration and tissue darkening. These four variables appeared to the right on the loading plots (Figure 2a,b, Figure 3a, Figure 4a and Figure 5). When analyzed, the variables cooking discoloration (Cook Dec and Cook Apr), losses during storage (Losses), stem branching (Branching), total amino acids (Aminoacid), crude protein (Crude) and pure protein (Pure) were also all expressed in this cluster to the right of the loading plot (Figure 2b and Figure 3a), as was the strength of the *Phytophtora* attack (Phyt) (Figure 4).

The second cluster consisted of the two core variables; dry matter and Quality indices (K-index). These two variables were expressed to the left along the first component (Figure 2a,b, Figure 3a, Figure 4a and Figure 5). When analyzed, the variables; ascorbic acid (Ascorbic ac), taste (Taste Dec and Taste Apr), EAA-indices (EAA-index) and relative proportion of pure protein (Rel. Pure) were also expressed to the left along the first component (Figure 2b and Figure 3a).

When excluding the eight core variables together with other variables closely correlated to the nitrogen factor, the relations between the remaining variables were more or less the same, although mirrored along the first principal component, with the exception of the variables yield and biocrystallization (comparing Figure 3a and Figure 6).

The variables yield, remaining yield (Remains), Yield after sorting (Sorted) and biocrystallization varied in their positions in the different PCA.

### 3.2. Differences between Field Trials

The differences between the UJ- and the K-trial were mainly expressed along the second component in Figure 2a. The variations among samples from the K-trial were located in the lower part of the PCA, whereas the samples from the UJ-trial were located in the upper part (Figure 2a).

The variation among samples from the K-trial were more related to the traits crystal and darkening of extract whereas the samples from the UJ-trial were more related to the traits yield and dry matter (Figure 2a).

The mean differences between biodynamic and conventional samples were slightly bigger in the K-trial, in comparison with the UJ-trial (Figure 2a).

### 3.3. Expressions of Farming Systems

Potato samples treated in a biodynamic farming system were consistently expressed more to the left in the PCA, in comparison with the corresponding samples treated conventionally (Figure 2a,b and Figure 3b).

When using the eight core variables on the data from both the K- and the UJ-trial 1971–1979, the biodynamic samples from the UJ-trial appeared more related to Quality-indices (K-index) and dry matter whereas the conventional samples were more related to free amino acids and yield (Figure 2a).

When using the overall mean values, from each of 22 different variables in the UJ-trial, the differences between treatments were expressed along the second principal component (Figure 2b). Biodynamic samples were here more associated to ascorbic acid and conventional samples more related to crude protein (Figure 2b).

When using the annual mean values, from the same 22 variables used in the UJ-trial 1971–1976, samples from 1972 and 1975 differed by being expressed more in the upper part, and slightly more to the right, in the PCA, in relation to other samples of the same treatment (Figure 3b). During these two seasons, the differences between biodynamic and conventional samples were located almost on a left to right line (Figure 3b). Concerning the other four years, the biodynamic samples were still located more to the left, but also higher, in comparison with the conventional samples (Figure 3b). Potato samples grown in the conventional system expressed a variation more related to the traits extract decomposition, amounts of free amino acids and tissue darkening **(Figure 3)**. The biodynamic samples showed a closer relation to the traits K-index, biocrystallization and relative amount of pure protein (Figure 3).

### 3.4. Type and Amount of Manure

Potato samples, from the K-trial, made into manure with biodynamic compost were consistently expressed to the left in the PCA plot, when compared with samples made into manure with inorganic fertilizers (Figure 4b). In 18 out of 20 seasons expressed in Figure 4b, the conventional samples were also expressed higher along the second component (Figure 4b). The biodynamic samples indicated a variation more related to Quality-indices, (K-index), dry matter and yield, while the orientation of the conventional samples showed a closer relation to free amino acid, extract discoloration and *Phytophtora* attack (Figure 4).

According to the PCA with potato samples from the additional UJ-trial, the two farming systems reacted quite differently on increasing amounts of manures (Figure 5). Potatoes from the biodynamic system exhibited a rather small variation between samples manured with increasing amounts of compost, forming a cluster in the lower left part of the PCA plot (Figure 5).

Increased amounts of compost, up to 252 kg N/ ha, were related to changes in the component score towards the variables yield, storage loss and darkening of tuber tissue (Figure 5). Potato samples given 315 kg N/ha of compost exhibited component scores more related to extract decomposition and free amino acids (Figure 5). Potatoes from the conventional system exhibited stronger, almost linear changes in the component scores when the amounts of inorganic fertilizers increased (Figure 5). An increase in the amount of inorganic fertilizers moved the component scores of the potato samples towards the traits of extract decomposition, darkening of tissue or discoloration of extract (Figure 5).

### 3.5. Influences of Crop Rotation

Using only mean values, potato samples originating from different crop rotation exhibited differences in location mainly along the second principal component (Figure 2b), while when using the annual values on the same material, there was also a difference along the first principal component (Figure 3b). Potato samples grown after barley were, with few exceptions, expressed higher and more to the left in the score plot, in comparison with samples grown after ley (Figure 2a,b and Figure 3b).

When grown after ley, the potato samples exhibited component scores more related to free amino acids than potatoes grown after barley (Figure 2a,b and Figure 3b).

During 1971, the first year of the UJ-trial, differences between samples from the different crop rotations were small in the biodynamic farming system but not in the conventional system (Figure 3b). The differences between potatoes from the two biodynamic crop rotations grew bigger during 1972 and 1973, as the rotation was implemented and then remained at the same level during the second rotation (Figure 3b). The differences between the two conventional samples did not show this development during the first six years of the UJ-trial (Figure 3b).

The differences, depending on preceding crop, were in average bigger in the biodynamic system, in comparison with the conventional, especially when using eight or 11 variables in the PCA-plot (Figure 2a and Figure 3b). The distance between the component scores from biodynamic and conventional samples were smaller when ley was included in the crop rotation, in comparison when it was not included (Figure 2a,b).

### 3.6. Interactions with Seasons and Cultivars

In the UJ-trial, 1971–1976, potatoes harvested in 1972 and 1975 were outliers in the PCA-plots. This was more obvious concerning the conventional samples than concerning the biodynamic samples (Figure 3b and Figure 6).

In the K-trial, 1970–1989, the seasons of 1972, 1973, 1985 and, to a certain degree, also the seasons of 1986 and 1989 were outliers in the PCA plot (Figure 4b).

Potato samples treated according to conventional principles exhibited in general a higher variance between seasons, that is a bigger distance between the component scores from different years, in comparison with samples treated biodynamically (Figure 3b, Figure 4b and Figure 6).

In the UJ-trial, differences between conventional and biodynamical samples were big in 1972 and small in 1976 (Figure 3b and Figure 6). In the K-trial, samples from 1971, 1974 and 1984 exhibited big differences between manure systems, whereas the differences were smaller between samples from 1972, 1973, 1975, 1976 and 1985 (Figure 4b). Samples from the K-trial, harvested in 1974 exhibited big differences in the component scores; the conventional treatment was located furthest to the right in the loading plot, while the biodynamic treatment was located at the lowest position in the plot (Figure 4b). Differences between manure system and seasons were expressed more along the first component concerning the cultivars King Edward and Bellona, while the differences between treatments were more related to the second component concerning the cultivars Grata and Provita (Figure 4b).

### 3.7. Differences Depending on the Variables Used in the PCA

Figure 6 displayed a PCA on a similar data set as in Figure 3, but the eight core variables and the other variables measuring the amounts of nitrogenous compounds were excluded from the data set. The differences between treatments were consistent also in Figure 6. The conventional samples were here located more to the left and higher up in the PCA plot, in comparison with the corresponding biodynamic samples (Figure 6). In comparison with Figure 3b, the potato samples in Figure 6, especially the two conventional samples, displayed a stronger relation to the yield and the cooking discoloration variables.

## 4. Discussion

All 29 potato harvest occasions included in the principal component analyses indicated a similar pattern: potato samples cultivated according to conventional principles exhibited a variance more related to higher amounts of crude protein, higher yield, more cooking discoloration, and faster extract decomposition. Potatoes treated in a biodynamic way were more related to traits such as higher Quality- and EAA-indices, higher dry matter content, better taste quality, higher relative proportion of pure protein and higher biocrystallization scores in relation to corresponding conventional samples.

This pattern was consistent, despite the differences in design between the K-trial and the UJ-trial. The K-trial was designed to give approximately the same yield levels among the manure treatments. The amounts of nitrogen given to the crop were different in each manure system. The UJ-trial was designed to supply each crop with approximately the same amounts of total nitrogen, regardless of type of fertilizer.

Although similar patterns were expressed in all PCA, the differences between biodynamic and conventional potato samples were not constant. It varied depending on crop rotation, season, and cultivar used. This variation needs to be taken into a deeper consideration before any general conclusions can be made out of the results presented here.

### 4.1. Principal Component Analysis

PCA is an important tool to analyze complex data sets. However, making significant statements out of the results from a PCA is difficult. Each PCA plot is unique. A small change in the matrix of data can change the plot dramatically. A thorough discussion on the results from the PCA is needed, if possible complemented with other statistical analyses, for example ANOVA, general linear model GLM or different measures of correlation.

The results from the PCA presented here were in accordance with the classical statistical evaluation presented in the Swedish report from the UJ-trial in Järna [38]. According to that evaluation, conventional potatoes grown in a crop rotation without ley significantly (*p* < 0.001) differed from the corresponding biodynamic samples by higher yield, higher amounts of crude and pure protein, stronger discoloration of tissues and extracts, faster extract decomposition, lower content of dry matter, lower amounts of ascorbic acid and lower values of both quality indices according to Pettersson and biocrystallization [38]. Comparing samples from a crop rotation with ley slightly decreased the differences between conventional and biodynamic samples. Here, conventional potatoes grown in a crop rotation with ley, significantly (*p* < 0.001), differed from the corresponding biodynamic samples by lower content of dry matter, higher amounts of crude and pure protein, lower amounts of ascorbic acid and lower values on biocrystallization. With a lower level of significance (*p* <0.05), conventional samples grown in a crop rotation with ley differed from the corresponding biodynamic samples by higher yield, stronger discoloration of tissues and extracts, faster extract decomposition and lower values of both quality indices according to Pettersson and biocrystallization [38].

### 4.2. Selection of Analyses

During the first half of the K-trial, analyses expressing differences were kept, while the others were left out. As a consequence, the analyses used came to mirror differences between treatments rather than giving a description of the actual potato crop. Such analysis, kept in the K-trial and used also in the UJ-trial, were biocrystallization, dry matter content, Quality indices (K-index), yield, dry matter content, extract decomposition, free amino acids, extract discoloration and tissue darkening.

Most of these eight core variables were strongly related to the amounts of easy soluble nitrogen available to the crop. In the statistical evaluation of the UJ-trial, there was a strong correlation (*p* < 0.001) between increasing amounts of crude protein and lower EAA-indices, lower amounts of ascorbic acid, lower dry matter content, lower taste scores, higher cooking discoloration, faster extract decomposition and faster darkening of potato extract or of potato tissue [38]. Similar correlations between these variables were found in the K-trial [35].

The core variables extract decomposition, free amino acids, extract discoloration and tissue darkening exhibited similar variance. This fact constituted some of the patterns along the first principal component displayed in most PCA plots. To a certain degree, this limits the value of the analyses presented in this paper, but taking the eight core variables away from the PCA did, however, not diminish the consistency in the differences between the treatments.

A wider distribution of analyses expressing different aspect of potato quality, concerning both primary and secondary metabolites, would, without doubt, have increased the information possible to extract with PCA from these two field trials.

### 4.3. Yield Levels and Crop Quality

The optimal amounts of manure, on the location of the UJ-trial, according to Figure 6, can be assumed to around 250 kg N/ha, of composted cow manure, or approximately 100 kg N/ha, applied as inorganic fertilizers. This indicates that the amounts given in the regular UJ-trial were low concerning composted manure and slightly high concerning inorganic fertilizers. The amounts of manure given in the UJ-trial were determined according to common practice at that period. In the biodynamic system, the amount of compost available is usually limited by the number of animals on the farm. The amounts of compost given in both the K-trial and the UJ-trial were higher than common practice. This was done in order to be more comparable with the conventional system.

A challenge to every farmer is to combine high yields with high crop quality. The results presented here indicate that the conventional farming system provides us with crops of higher yield but lower quality in comparison with crops from the biodynamic system, whereas the measurement of yield is simple, and it is commonly accepted that there are different opinions concerning what is meant by high crop quality [2,3,10,48].

Most authors agree in the opinion that food quality is too complex to be reduced into a single value. Three general concepts; vital qualities, organic integrity and true nature of a crop have been suggested as important to develop further, at least within the OA movement, in order to find adequate methods to assess crop quality [48]. So far, no single method has been found to express these three concepts in one score. Neither has one method been confirmed to express differences consistently between farming systems. This does not mean that such differences do not exist. Differences between farming systems might be on structural level too high to be captured in one measurement.

With this paper, we tried to bring the results from separate analyses together into a number of plots or pictures. Another way would be to develop and use some sort of relative values or indices, such as the Quality-indices or the EAA-indices. A third possible way would be to develop methods that assess food quality on a higher structural level [49].

### 4.4. The Biocrystallization Method

Biocrystallization, also called sensitive cupper chloride crystallization, was developed to assess structural traits of a crop. This is not the place to describe or validate this method. This has been done elsewhere [45,46,50,51]. In the UJ- and K-trial, the results from the biocrystallization were reduced to values based on the number of disturbances in the structure of the crystallization pattern. In the results presented here, few disturbances in the pattern gave high scores.

The PCA plots did not display a consistent value of the component scores concerning biocrystallization. When the first component in the PCA was dominated by traits related to the nitrogen factor, the crystallization value was situated, more or less alone, in the lower part of the second component. This could indicate that this value expressed another aspect of crop quality, diverted from the nitrogen factor. When a larger number of different parameters were used, the biocrystallization score was expressed opposite to crude protein but close to ascorbic acid and relative proportion of pure protein. In a PCA without the variables positively related to the amounts of nitrogen, the position of the component score of biocrystallization indicated a positive relation to the amounts of ascorbic acid and a negative to storage losses and discoloration during cooking of the potatoes. If the biocrystallization score was an expression of the structural quality of the potatoes, the results presented here can indicate that increasing amounts of inorganic nitrogen decreases the structural quality of the potatoes. However, this still needs to be documented more in detail.

### 4.5. Indices and Relative Values as Expressions of Crop Quality

The Quality indices according to Pettersson, (K-index) did not bring anything new into the PCA plot. It was always located opposite to the traits it was calculated from. The three variables; extract decomposition, free amino acids and extract discoloration, were too close to each other in their variation to express any broader concept of quality when put together into one indices.

The EAA-indices, that is, the relative, structural, composition of amino acids, appeared opposite to the amounts of amino acids in the principal component analyses. In addition, the amounts of crude and pure protein appeared opposite to the relative proportion of pure protein. In both these examples, the absolute amount of a compound exhibited a variation opposite to the relative proportion of this compound. Furthermore, in both these examples, increasing absolute amounts of free amino acids, crude protein, and even pure protein exhibited a variation positively related to discoloration and decomposition. A high amount of a desirable substance might not always be a sign of good potato quality. This does not mean that the lowest amounts of amino acids in a crop would be preferable. Instead, it is an optimal amount, both absolute and relative, that needs to be sought for. In the relative composition of the single substances, a higher level of crop quality might be expressed.

### 4.6. Type and Amount of Fertilizer

Is it the type of fertilizer or is it the amount that influences the quality of the potatoes the most? The results presented here indicated, not so surprisingly, that both the type and the amount of fertilizer influenced the quality of the potatoes. There was a consistent difference in the component score if using biodynamic compost or using inorganic fertilizers. Increasing the amount of compost did not initiate component scores close to the scores reached when using inorganic fertilizers. On the contrary, the scores changed relatively little when increasing the amounts of compost from zero to 315 kg N/ha. Increasing the amounts of inorganic fertilizers to potatoes changed the component scores radically. The results presented here indicated that in a Scandinavian climate increased amounts of inorganic fertilizers faster contributed to decreasing potato quality than did increased amounts of compost.

### 4.7. Influences of Crop Rotation

In the biodynamic system, potatoes grown after ley exhibited component scores closer to the traits of free amino acids and extract decomposition, in comparison with potatoes grown after barley. In the conventional system, samples with ley as preceding crop were more related to the trait yield. Potatoes grown after ley were manured with 20 kg N/ha less than the potatoes grown after barley. The difference in component score between samples from different crop rotation can therefore not depend on the actual amounts of nitrogen given to the crop that year. The reason to the differences is more likely to be found in the nitrogen assimilated by the clover in the ley the year before. Apparently, the amount of nitrogen mineralized in the soil after ley exceeded the decrease in the nitrogen supplied by manures or fertilizers. The stronger increase in yield seen among samples from the conventional system indicate that 120 kg N/ha given as mineral fertilizer to potatoes after barley was too low to reach the highest yield levels. The ley treated biodynamical had a slightly higher proportion of clover in the ley, in comparison with the corresponding plots treated conventional [38]. This can account for some of the observed enlarged differences between samples from different crop rotations in the biodynamic system. If barley was a poorer preceding crop to potatoes in the biodynamic system, than in the conventional, this might also have contributed to the observed differences between the farming systems.

### 4.8. Differences between Seasons and Cultivars

The weather conditions during the different seasons had a major impact on the results. For some of the years, there was hardly any difference between samples regardless of treatment. In general, the conventional system exhibited a higher variation between seasons. One reason for this might be the fact that the mineral fertilizer needed to be liquefied by rain before it could reach the plant roots. The biodynamic system was more dependent on the temperature factor, as a certain soil temperature was needed to initialize the mineralization processes. During cold and dry seasons, such as 1976, the differences between biodynamic and conventional systems were small. A rainy and cold season, such as 1974, led to bigger differences between the systems. This was probably due to the fact that the rain made the inorganic fertilizers available for the plants, but the low temperature decreased the rate of plant development. In the conventional system, this led to a relative higher yield of small tubers with a relative higher content of free amino acids. These examples also stress the fact that a comparison between organic and conventional systems always must be considered on the bases of the actual weather conditions.

The difference between the biodynamic and the conventional samples were also dependant on the cultivar used. In the UJ-trial, the differences between systems were bigger when the cultivar Bintje was used in comparison with when the cultivar Grata was used. In the K-trial, the cultivar King Edward expressed bigger differences between the systems than did the cultivars Grata, Bellona or Provita. The differences between the systems were also expressed differently between parameters. For example in the K-trial, the trait extract decomposition expressed bigger differences between manure systems when the cultivar Grata was used in comparison to periods when the cultivars King Edward, Bellona and Provita were used [35].

### 4.9. Comparing Farming Systems and Long Term Field Trials

The first factor needed when comparing farming systems is time. Agricultural systems are developing very slowly. The soil conditions needs time to establish. The nine year trial period of the UJ-trial must be considered as short, but, even after 33 years, the soil fertility were still developing in the K- trial [35].

The second factor of importance is to compare systems and not treatments. This means that crop rotations, cultivars, and some other measures must differ between the compared systems. In this sense, the K-trial was more an experiment comparing different manuring strategies, while the UJ-trial came closer to comparing systems. However, the crop rotations used was much too short to represent at least the biodynamic system. In addition, the cultivars used were the same in both systems. They were not chosen in accordance with the prevailing situation in the different systems. Conventional and biodynamic systems need cultivars of different character in order to express their full potential.

The third factor deals with the parameters used to assess the crop. It is necessary to use analyses together expressing the substantial, structural, functional and nutritional qualities of a crop [48,49,50,51]. The results from the K- and UJ-trial reflect some of the substantial and structural qualities of the potato crop but lack direct analyses of the nutritional qualities of the products. The methods used to assess the potatoes from the two field trials focus mainly on the importance of the nitrogen factor.

## 5. Conclusions

Samples treated according to conventional principles consistently exhibited a variation more positively related to amounts of crude protein, yield, cooking discoloration and extract decomposition. Samples treated in a biodynamic way were more positively related to traits such as Quality- and EAA-indices, dry matter content, taste quality, relative proportion of pure protein, and biocrystallization value. Distinctions between seasons, crop rotations and cultivars used were sometimes more significant than differences between farming systems. In both the conventional and the biodynamic system, the potato crop exhibited better quality traits grown after barley compared to when grown directly after ley.
